# The Benefits and Respective Side-Effects of PE Therapy for Intractable Kawasaki Disease

**DOI:** 10.3390/jcm10051062

**Published:** 2021-03-04

**Authors:** Masaaki Mori, Susumu Yamazaki, Takuya Naruto

**Affiliations:** Department of Lifetime Clinical Immunology, Graduate School of Medical and Dental Sciences, Tokyo Medical and Dental University, Tokyo 113-8510, Japan; susumu@juntendo.ac.jp (S.Y.); tnaruto.lci@gmail.com (T.N.)

**Keywords:** coronary artery lesions (CALs), cytokine, early intervention, intravenous immune-globulin refractory, plasma exchange (PE)

## Abstract

Kawasaki disease (KD) is a vasculitis syndrome that frequently develops coronary artery lesions (CALs). In the treatment of KD, the utility of high-dose intravenous immuno-globulin (IVIG) therapy has already been clarified, and it has been established as the first-line treatment method. However, since approximately 10% of patients are refractory to this IVIG therapy and 2.6% of all patients have coronary sequelae, 500 children with KD still remain every year in Japan. In this disease, it is necessary to calm inflammation within 10 days of onset in order to suppress CALs caused by a large amount of persistent inflammatory cytokines. Indeed, the early suppression of inflammation is an effective means of suppressing the onset of CALs. Here, we describe the pathophysiology of Kawasaki disease and plasma exchange (PE), which is a therapeutic method that can calm the hyper-cytokine state of this disease. The treatment result of PE for IVIG-refractory Kawasaki disease is outstanding, and an extremely large effect can be expected if it can be started before the appearance of CALs. It seems that it should always be considered as one of the powerful additional treatments in the future.

## 1. Introduction

Kawasaki disease (KD) is a vasculitis syndrome that frequently develops coronary artery lesions (CALs) [1]. In this disease, it is necessary to suppress inflammation within 10 days of onset in order to suppress CALs caused by a large amount of persistent inflammatory cytokines. Indeed, the early suppression of inflammation is an effective means of suppressing the onset of CALs.

In this review, we first describe (1) the epidemiology, diagnosis, diagnostic tests, and treatment strategies of Kawasaki disease; (2) practical aspects of plasma exchange (PE) therapy in KD; (3) indications for PE therapy; (4) the utility of PE as an additional treatment; and (5) we outline the evaluation of evidence and insurance indications for PE therapy. Finally, we summarize the pathophysiology of KD and plasma exchange, which is a therapeutic method that can calm the hyper-cytokine state of this disease.

## 2. The Whole Picture of Kawasaki Disease in Japan

### 2.1. Epidemiology

The incidence of KD has been increasing every year. The results of the 25th nationwide survey showed an extremely high incidence rate of 359.1 per 100,000 population aged 0–4 years in 2018, and the annual number of patients reached a record high of 17,364 [2]. In the treatment of KD, the utility of high-dose intravenous immuno-globulin (IVIG) therapy has already been clarified, and it has been established as the first-line treatment method [3,4]. However, the refractory rate of the initial dose in pediatric patients was 19.7% (Table 1), and even after several doses, 5–10% of children were refractory to the treatment. Moreover, CALs, sequelae of Kawasaki disease, still occur in 500–800 cases annually, which is the main problem in the treatment of this disease.

### 2.2. Diagnosis

The diagnosis of this disease is currently being made in accordance with the sixth revised edition of the diagnostic manual (Table 2) [5]. The natural history of Kawasaki disease is characterized by the overlapping occurrence of acute symptoms over the course of time. By days three to seven, the clinical picture is complete with the presence of vasculitis symptoms such as fever, cervical lymphadenopathy, bulbar conjunctival hyperemia, strawberry tongue, redness and cracking of the lips, hard edema of the limbs, and redness of the tips of the digits. Abnormal redness of the BCG (Bacillus Calmette-Guerin) vaccination site before or after the onset of fever is also a characteristic feature of the disease. In patients with subsequent CALs, “increased brightness” of coronary arteries is observed on echocardiography starting around day 10, and coronary artery dilation and aneurysm formation are detected between days 10 and 14.

### 2.3. Diagnostic Tests

Characteristics of hematological findings in “hypercytokinemia” include (1) markedly increased leukocyte count (normally ≥ 10,000/μL) and elevated neutrophil fraction; (2) elevated inflammatory markers (CRP(C-reactive protein)/ESR(erythrocyte sedimentation rate)/serum amyloid A); (3) hyper-activation of the congealing fibrinogenolysis system (PT(prothrombin time)/APTT(activated partial thromboplastin time)/fibrin degradation products (FDP-E/D-dimer)) and decreased serum albumin level; and (4) elevated cytokine-induced protein (urinary β2-microglobulin/serum ferritin). However, for unknown reasons, there are not many patients who experience an extremely severe cytokine storm that is destructive to tissues and organs as seen in other hypercytokinemic disease groups.

### 2.4. Treatment Strategies

The direction of treatment is to suppress vasculitis before the development of CALs, rather than only treating the various clinical symptoms seen in the acute phase. Therefore, the following issues must be addressed in practice: (1) how to suppress inflammation by days 7 to 10; (2) how to treat severe cases in which CALs develop by day 7; and (3) how to treat cases in which inflammation persists until day 10 or later. The principle of treatment is the early suppression of inflammation, and general cases should be treated with (1) high-dose gamma-globulin IVIG therapy; (2) anticoagulation therapy with aspirin; (3) thrombolytic therapy for aneurysm formation and measures to prevent myocardial infarction as well as peripheral arterial disorders; and (4) measures to prevent non-cardiovascular complications (meningitis, encephalopathy, DIC(disseminated intravascular coagulation), etc.).

## 3. Practical Aspects of PE Therapy in Kawasaki Disease

The basic principle of PE is that a membrane-type plasma separator is used, and filtration is performed by transmembrane pressure (TMP). Blood is drawn via vascular access (VA) and passed through a membrane-type plasma separator, where the blood is separated into blood cell and plasma components, and the plasma components are then discarded. Subsequently, is the remaining components are supplemented with the same volume of replacement fluid, and the fluid is transferred back into the body.

When PE is performed in children with a small body size and low circulating blood volume, it is necessary to ensure reliable VA, reduce the priming volume (PV), prevent hypothermia, use anticoagulants, and provide safe sedation [6]. PE has been implemented under ICU(intensive Care Unit) or PICU(pediatric ICU) management, with a target PE volume of approximately 1 to 1.5 circulating plasma volumes (circulating blood volume (mL) = body weight (kg)/13 × (100 − hematocrit (%)/100) × 1000). The standard treatment period is three days, but this can be extended to a maximum of five days if fever and inflammatory response do not resolve (Table 3).

### 3.1. Selection of Blood Circuit and Setting of Equipment

Since the PV is relatively large compared to the circulating blood volume, even if a low-volume blood circuit is used, close attention must be paid to the decrease in parameters and changes in vital signs at the start of priming. In general, the PE machines and blood circuits should be selected and prepared to ensure that the PV is less than 10% (8 mL/kg) of the circulating blood volume. However, since this is difficult to implement in neonates and infants, it is necessary to fill the circuit with blood products such as RCC(red cells concentrates) or FFP(fresh frozen plasm) at the beginning of treatment if the PV is higher than 8 mL/kg to prevent initial changes in parameters or vital signs (initial drop) due to hemodilution. The only plasma separator available in Japan is the Plasmaflo™ (Asahi Kasei Medical, Frankfurt, Germany) OP-02W, and the PV, including plasma, is slightly less than 60 mL when PE is performed using this separator.

When performing PE, Q_B_ should be set at 20–30 mL/min. It is not necessary to increase Q_B_ more than necessary, but it is not desirable to set it too low because it will prolong the time taken for blood to pass through the circuit. Since PE is a treatment that utilizes filtration as the operating principle, there is theoretically little reduction in treatment efficiency due to decreased Q_B_. Therefore, treatment should be performed at the lowest Q_B_ (20–30 mL/min) that does not result in intra-circuit coagulation, and consideration should be given to the stability of circulatory dynamics. Quarantine plasma (Q_P_) should be 20% of Q_B_ as a maximum, and attention should be paid to the increase in TMP.

### 3.2. Measures against Hypothermia

Children are naturally prone to hypothermia because of their large body surface area for their body weight, high respiratory rate, high minute ventilation volume for their body weight, low subcutaneous fat, and high percutaneous evaporation due to their thin and immature skin. In addition, during priming, the ratio of PV to circulating blood volume is higher, making it more prone to causing hypothermia. The heating function of the PE machine alone is often not sufficient to prevent a drop in body temperature, and it may be necessary to warm the circuit itself or to warm the pediatric patient with an infant warmer.

### 3.3. Anticoagulants

Children may require more anticoagulants than adults due to the inability to obtain a sufficient priming blood volume due to their small body size and the large capacities of the circuit and membrane areas relative to their body size. Although heparin sodium is generally used, nafamostat mesilate may be used if a patient has a bleeding tendency or thrombocytopenia. Anticoagulation is monitored by measuring activated clotting time (ACT), which can be done easily and quickly at the bedside, and detailed adjustments can be made to prevent intra-circuit coagulation. The target ACT is 180–250 s.

### 3.4. Sedation during PE Implementation

Since blood flow and priming blood volume are very high and flexion and removal of the catheter can be very dangerous in infant and young child patients, they require reliable sedation for the safe implementation of PE. PE under tracheal intubation and ventilatory control should be considered in some cases, and since it is essential to respond quickly to sudden changes in vital signs during priming, management in the ICU or PICU is desirable whenever possible.

### 3.5. Complications of PE

Complications from securing VA (pneumothorax, vascular injury, and catheter-associated infection), reactions to anticoagulants and replacement fluids (anaphylaxis, bleeding tendency, and hypocapnia), and complications from priming itself (hypotension, hypovolemic shock, and hypothermia) may occur. Changes in vital signs, especially at the start of priming, must be closely monitored.

## 4. Indications for PE Therapy

Before the advent of infliximab (IFX), IVIG therapy, the standard treatment, was initiated promptly after diagnosis of Kawasaki disease in the facility, and patients who did not respond to the initial IVIG were treated with additional IVIG. In cases where no improvement is observed, PE is often selected for the IVIG-refractory patients. All clinical studies were conducted in accordance with the Declaration of Helsinki, with the informed consent of the participants, and approved by the Ethics Committee of the respective facilities.

IVIG-refractory cases are determined when the following items are met within 48 h of the end of IVIG administration:

(1) If the patient has a fever of 38 °C or higher at least once a day.
(2) If fractional change (FC) of at least one of the three inflammatory markers (leukocyte count, neutrophil count, or CRP) in blood tests does not improve.

The use of FC for severity assessment is a method that we proposed to determine the severity of disease to identify IVIG-refractory cases and the risk group for complications with CALs [7]. This was a retrospective study based on clinical data of 193 patients with Kawasaki disease treated with IVIG, with a particular focus on the changes in hematological and biochemical laboratory data before and after IVIG treatment. The rate of change of each laboratory datum was defined as fractional change (FC) as follows:

FC = (laboratory data 48 h after IVIG administration) − (laboratory data before IVIG administration)/(laboratory data before IVIG administration)

After dividing 193 patients into the group with normal coronary arteries (*n* = 169) or the group with CALs (*n* = 24), FCs of leukocyte count, neutrophil count, platelet count, CRP, and albumin were examined. Significant differences were found in the FCs of leukocyte count, neutrophil count, and CRP level. While 1.8%, 3.6%, and 4.7% of patients in the normal coronary artery group had positive FCs (trend toward worsening), the rates were higher in the CALs group at 87.5%, 79.1%, and 66.7%. There was no significant difference in platelet count or albumin. We conclude that patients with a positive FC for either leukocyte count, neutrophil count, or CRP level are severe cases with a high risk of coronary complications, and that the obviously high incidence of coronary complications in IVIG-refractory cases is useful information in establishing criteria for the introduction of additional treatment (Table 4).

## 5. Utility of PE as an Additional Treatment

**Figure 1:**
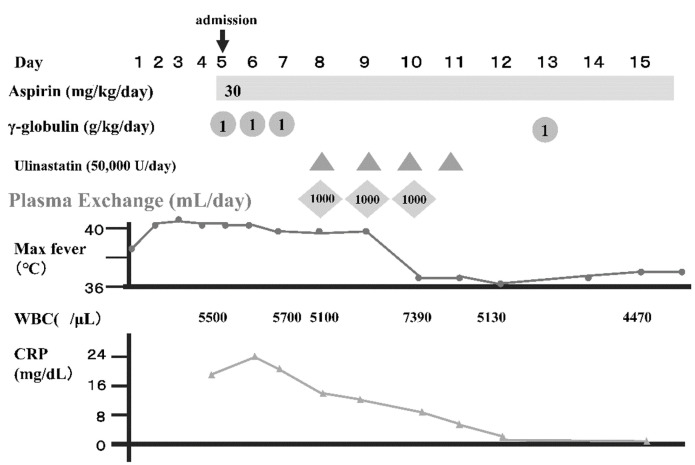
Clinical course of a 2-year-old boy with PE treatment.

Figure 1 shows the typical course of PE in an IVIG-refractory patient. It is clear that fever resolved quickly and that the various clinical symptoms and laboratory findings normalized over the course of treatment.

**Figure 2:**
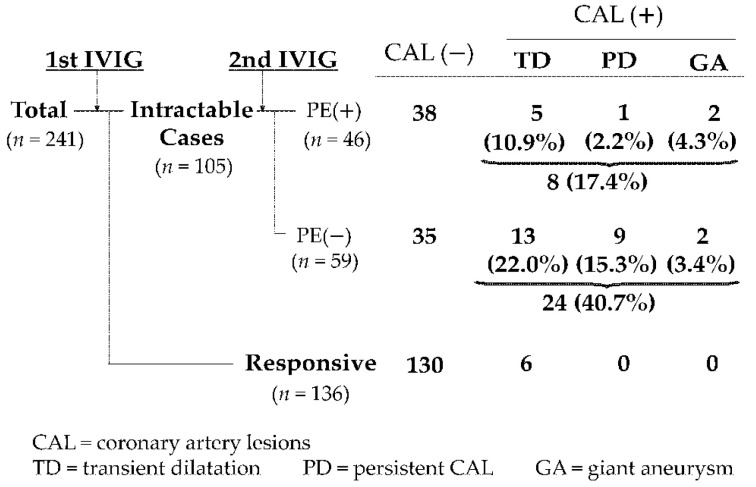
Preventive effect of PE on CALs in IVIG-refractory patients.

In our 2004 report, we investigated the safety and preventive effect of PE on CALs in IVIG-refractory patients (Figure 2) [8,9]. The 105 patients who were refractory to second IVIG treatment were divided into two groups: those who received PE therapy (PE(+) group: 46 patients) and those who received additional IVIG (PE(−) group: 59 patients), and their incidence of CALs in the acute phase was compared and examined. The patients were divided into three groups: transient dilatation (TD), in which coronary artery dilation was found but normalized within 30 days of onset; persistent dilatation (PD), in which dilation remained beyond 30 days; and giant aneurysms (GA), in which the aneurysm was larger than 8 mm in diameter. In the PE(−) group, CALs were found in 24 (40.7%) of 59 patients (13 TD, 9 PD, and 2 GA), whereas in the PE(+) group, CALs were found in only 8 (17.4%) of 46 patients (5 TD, 1 PD, and 2 GA), showing a sufficiently significant difference (*p* = 0.022). Multivariate analysis revealed that PE therapy significantly reduced the incidence of CALs compared with additional IVIG therapy, with an odds ratio of 0.052 (*p* = 0.0012) (Table 5).

We further investigated the long-term course of CALs (>1 year) by greatly increasing the number of subjects [10]. Since the study was conducted over a period of more than 10 years, there were differences in the methods of administration of IVIG as initial treatment, additional treatment, and day of PE initiation, but if PE could be started before the coronary arteries began to dilate, the residual sequelae rate was 0% (0/105), which is a very favorable result. In patients whose dilation had already started before PE, the residual sequelae rate was 30% (6/20), showing a statistically significant difference (*p* < 0.001). Of five patients who were left with large aneurysms as sequelae, four had obvious aneurysm formation before PE (the remaining patient also had dilation), suggesting that sequelae could not have been avoided even if treatment other than PE had been chosen as additional treatment.

In addition, it has recently been shown that a stepped-care approach of infliximab (IFX) combined with PE can suppress CAL formation more potently in Kawasaki disease that is refractory to additional IVIG therapy [11]. IFX treatment was additionally provided to 76 IVIG-refractory patients, and PE was performed as a salvage treatment to 6 IFX-refractory patients. In all cases, fever and other clinical symptoms were improved, and significant inflammatory suppression was observed in laboratory values, resulting in the suppression of coronary artery disorders and complete recovery in all patients. In addition to IFX-refractory patients, there were patients who were converted to PE early without additional IVIG or INF administration in cases of late diagnosis, severe complications such as impaired consciousness or decreased cardiac function, or coronary artery changes that had already begun at the time of diagnosis. Since the ultimate goal of the treatment of Kawasaki disease is to suppress the formation of CALs, it has been suggested that this goal may be achieved satisfactorily by a stepped approach and rapid therapy of (IVIG + IFX + PE).

## 6. Evaluation of Evidence and Insurance Indications for PE Therapy

Most of the efficacy studies have been case reports. Joh was the first to mention the possibility of PE for Kawasaki disease [12], and Takagi et al. reported that a 4-year-old girl who was refractory to IVIG was treated with PE for 3 days and that the inflammation was successfully suppressed with no coronary complications [13]. There have been no reports based on prospective studies and only two reports of retrospective studies, including the one we previously reported. Villain et al. performed a comparative study of 20 patients treated with PE or IVIG within 15 days of disease onset [14]. PE was performed in eight patients (seven had exchange transfusions and only one had plasmapheresis), and IVIG was performed in twelve patients. Although sufficient statistical analysis was not performed, the authors reported that no coronary lesions developed in any of the patients and that there were no safety issues.

In addition, since April 2012, PE for Kawasaki disease has been covered by insurance and can be claimed up to six times per case when immunoglobulin therapy, pulse steroid therapy, or therapy with neutrophil elastase inhibitors is ineffective or not indicated in Japan.

## 7. Conclusions

PE, one of the blood purification techniques, is a treatment method in which blood is separated into blood cell and plasma components and replaced with equal amounts of fresh frozen plasma (FFP), albumin, or other substances. The aim is to remove pathological substances (autoantibodies, immune complexes, albumin-binding toxins, inflammatory cytokines, chemokines, etc.) contained in plasma. PE is performed in Kawasaki disease in order to directly remove inflammatory cytokines, chemokines, and proinflammatory substances from the circulating blood and to suppress inflammation in an early stage.

In Kawasaki disease, it is critical to respond to treatment before day 10, when CALs are most likely to develop, and it is well-known that early suppression of inflammation is directly related to the suppression of CALs. The treatment result of PE for IVIG-refractory Kawasaki disease is good, and if it can be started before the appearance of CALs, an extremely large effect can be expected. It seems that it should always be considered as one of the powerful additional treatments for intractable cases in the future.

## Figures and Tables

**Table 1:** First immunoglobulin therapy for Kawasaki disease (KD).

	Initial IVIG ^1^ Administration Rate	First IVIG ^1^ Refractory Rate
25th National Survey (2017–2018)	94.6% (30,784/32,528)	19.7% (6061/30,784)
24th National Survey (2015–2016)	93.5% (29,543/31,595)	17.8% (5250/29,543)
23rd National Survey (2013–2014)	92.6% (29,322/31,675)	17.1% (5024/29,322)
22nd National Survey (2011–2012)	91.4% (24,346/26,691)	17.0% (4150/24,346)
21st National Survey (2009–2010)	89.5% (21,247/23,730)	16.6% (3532/21,247)

^1^ IVIG: intravenous immunoglobulin.

**Table 2:** Main symptoms in the diagnostic guide of KD ^1^ (sixth revision).

1	High fever conjunctiva
2	Hyperemia of bilateral eyeball conjunctiva
3	Lips and oral findings: lip flush, strawberry tongue, diffuse redness of oral cavity and pharyngeal mucosa
4	Rash (including redness of BCG ^2^ inoculation marks)
5	Changes in extremities –Acute phase—rigid edema of the limbs and erythema on the palms and soles or fingertips –Recovery phase—membranous desquamation from fingertips
6	Non-suppurative cervical lymphadenopathy in the acute phase

^1^ This is a disease with five or more of the six main symptoms or four symptoms with coronary artery enlargement/aneurysm when other diseases have been discarded. ^2^ Bacillus Calmette-Guerin

**Table 3:** Method and procedure of plasma exchange (PE) therapy.

Machine	JUN-500 (Ube)
Circuit	JCH-11 s, 10 s (priming volume 30 mL)
Plasma separator(low membrane dimension)	Plasmaflo OP-02W (Asahi Medical, total circuit priming volume 79 mL)
Displacement liquid	5% albumin
Exchange volume	~1–1.5 plasma volume
Catheter	6–7 Fr 10 cm double-lumen catheter for pediatric hemodialysis (e.g., Gam Cath), mainly punctured and kept in patient’s external cervical vein or femoral vein (subclavian vein): Qb(blood flow rate) = 20–60 mL/min; Qs (replacement flow rate) = 300–600 mL/h
Anticoagulant	Mainly heparin sodium (one shot at the start of PE 15–30 U/kg, continuous 15–30 U/kg)
ACT (activated clotting time)	180–250 s (controlled amount of heparin)
Arterial line	Not necessary, blood pressure monitored by fits

**Table 4:** Effectiveness of fractional change.

	Group with Normal Coronary Arteries (*n* = 169)	Group with Coronary Artery Lesions (CALs) (*n* = 24)	*p* Value
FC (+) of leukocyte count	1.8%	87.5%	<0.001
FC (+) of neutrophil count	3.6%	79.1%	<0.001
FC (+) of CRP level	4.7%	66.7%	<0.001

CRP: C-reactive protein.

**Table 5:** PE therapy significantly reduced the incidence of CALs.

Multivariate Analysis	Odds Ratio (95% Confidence Interval)		*p* Value
Plasma exchange (−)	1	(0.007–0.244)	0.0004
(+)	0.052		

## Data Availability

The data that support the findings of this study are openly available in (repository name e.g., “figshare”) at http://doi.org/[doi] (accessed on 29 January 2021), reference number (reference number).
